# Stable Matching with Uncertain Linear Preferences

**DOI:** 10.1007/s00453-019-00650-0

**Published:** 2019-11-14

**Authors:** Haris Aziz, Péter Biró, Serge Gaspers, Ronald de Haan, Nicholas Mattei, Baharak Rastegari

**Affiliations:** 1 UNSW Sydney, Sydney, Australia; 2 Data61, Sydney, Australia; 3 Hungarian Academy of Sciences, Budapest, Hungary; 4 Corvinus University of Budapest, Budapest, Hungary; 5 ILLC, University of Amsterdam, Amsterdam, The Netherlands; 6 Tulane University, New Orleans, USA; 7 University of Southampton, Southampton, UK

**Keywords:** Stable matchings, Stable marriage problem, Uncertain preferences, NP-hard problems, Polynomial-time algorithms

## Abstract

We consider the two-sided stable matching setting in which there may be uncertainty about the agents’ preferences due to limited information or communication. We consider three models of uncertainty: (1) lottery model—for each agent, there is a probability distribution over linear preferences, (2) compact indifference model—for each agent, a weak preference order is specified and each linear order compatible with the weak order is equally likely and (3) joint probability model—there is a lottery over preference profiles. For each of the models, we study the computational complexity of computing the stability probability of a given matching as well as finding a matching with the highest probability of being stable. We also examine more restricted problems such as deciding whether a certainly stable matching exists. We find a rich complexity landscape for these problems, indicating that the form uncertainty takes is significant.

## Introduction

We consider a *Stable Marriage problem (SM)* in which there is a set of men and a set of women. Each man has a linear order over the women, and each woman has a linear order over the men. For the purpose of this paper we assume that the preference lists are complete, i.e., each agent finds each member of the opposite side acceptable.^1^ In the stable marriage problem, the goal is to compute a *stable matching*; a matching where no two agents prefer to be matched to each other rather than to be matched to their current partners. Unlike most of the literature on stable matching problems [13, 19, 27], we assume that men and women may have uncertainty in their preferences which can be captured by various probabilistic uncertainty models. We focus on *linear models* in which each possible deterministic preference profile is a set of linear orders.

Uncertainty in preferences could arise for a number of reasons, both practical or epistemological. For example, an agent could express a weak order because the agent did not invest enough time or effort to differentiate between potential matches and therefore one could assume that each linear extension of the weak order is equally likely; this maps to our *compact indifference model*. In many real applications, the ties are broken randomly with lotteries, e.g., in the school choice programs in New York and Boston as well as in centralized college admissions in Ireland. However, a central planner may also choose a matching that is optimal in some sense, without breaking the ties in the preference list. For instance, in Scotland they used to compute the maximum size (weakly) stable matching to allocate residents to hospitals [19]. Alternatively, there may be a cost associated with eliciting preferences from the agents, so a central planner may want to only obtain and provide a recommendation based on a subset of the complete orders [9, 24]. Another cause of uncertainty could be that agents are certain about preferences over other agents according to specific criteria but there may be a probability distribution corresponding to the weight placed on different criteria. This motivates our lottery and joint probability models. In the *lottery model*, the agents have *independent* probabilities over possible linear orders, i.e., each linear order may correspond to a different criterion. In the *joint probability model*, the probability distribution is over possible preference profiles.

Uncertainty in preferences has already been studied in voting [15] and for cooperative games [18]. Ehlers and Massó [10] considers many-to-one matching markets under a Bayesian setting. Similarly, in auction theory, it is standard to examine Bayesian settings in which there is a probability distribution over the types of agents. In the two-sided matching setting, when preferences are uncertain, a natural solution is finding a matching which has the highest probability of being stable. We consider this fundamental computational problem and its related variants for the three uncertainty models discussed above.

To illustrate the problem, we describe a simple example with four agents. We write $b~{\succ }_a c$ to say that agent *a* prefers *b* to *c* and assume the lottery model.

### Example 1

We have two men $m_1$ and $m_2$ and two women $w_1$ and $w_2$. Each agent assigns a probability to each strict preference ordering as follows. (i) $p(w_1 {\succ }_{m_1} w_2 )=0.4$ and $p(w_2 {\succ }_{m_1} w_1 )=0.6$ (ii) $p(w_1 {\succ }_{m_2} w_2 )=0.0$ and $p(w_2 {\succ }_{m_2} w_1 )=1.0$ (iii) $p(m_1 {\succ }_{w_1} m_2 )=1.0$ and $p(m_2 {\succ }_{w_1} m_1 )=0.0$ (iv) $p(m_1 {\succ }_{w_2} m_2 )=0.8$ and $p(m_2 {\succ }_{w_2} m_1 )=0.2$.

**Table 1:** Pairwise probabilities for the agents in Example 1

Men	Women
$$\begin{array}{rrr} m_1 &{} &{}p(w_1 {\succ }_{m_1} w_2 )=0.4 \\ &{} &{}p(w_2 {\succ }_{m_1} w_3 )=0.6 \\ &{} &{} \end{array}$$	$$\begin{array} {rrr} w_1 &{} &{} p(m_1 {\succ }_{w_1} m_2 )=1.0 \\ &{} &{} p(m_2 {\succ }_{w_1} m_3 )=0.0 \\ &{} &{} \end{array}$$
$$\begin{array}{rrr} m_2 &{} &{} p(w_1 {\succ }_{m_2} w_2 )=0.0 \\ &{} &{} p(w_2 {\succ }_{m_2} w_3 )=1.0 \\ &{} &{} \end{array}$$	$$\begin{array} {rrr} w_2 &{} &{} p(m_1 {\succ }_{w_2} m_2 )=0.8 \\ &{} &{} p(m_2 {\succ }_{w_2} m_3 )=0.2 \\ &{} &{} \end{array}$$

This setting admits two matchings that are stable with positive probability: $\mu _1 = \{(m_1,w_1), (m_2,w_2)\}$ and $\mu _2 = \{(m_1,w_2), (m_2,w_1)\}$. Notice that if each agent submits the preference list that s/he finds most likely to be true, then the setting admits a unique stable matching that is $\mu _2$. The probability of $\mu _2$ being stable, however, is 0.48 whereas the probability of $\mu _1$ being stable is 0.52 (Tables 1, 2).

**Table 2:** Stability probability for each matching in Example 1

	Matching	Stability probability
$\mu _1$	$$\begin{array}{c}\{(m_1,w_1), (m_2,w_2)\}\end{array}$$	0.52
$\mu _2$	$$\begin{array}{c}\{(m_1,w_2), (m_2,w_1)\}\end{array}$$	$$\begin{array}{c}0.48 \end{array}$$

### Uncertainty Models

In this article, we consider three different uncertainty models which assume that agents have *linear* preferences. In related work we have explored similar computational questions when agents define their uncertainty over *pairwise* preferences [3].

- *Lottery Model* For each agent, we are given a probability distribution over strict preference lists.
- *Compact Indifference Model* Each agent reports a single weak preference list that allows for ties. Each complete linear order extension of this weak order is assumed to be equally likely.
- *Joint Probability Model* A probability distribution over preference profiles is specified.

Note that for the Lottery Model and the Joint Probability Model the representation of the input preferences can be exponentially large (in the number of agents). However, in settings where similar models of uncertainty are used, including resident matching [9] and voting [15], a limited amount of uncertainty (i.e. small supports) is commonly expected and observed in real-world data [21, 22]. Consequently, we consider special cases when the uncertainty is bounded in certain natural ways, including the existence of only a small number of uncertain preferences and/or uncertainty on only one side of the market.

Observe that the compact indifference model can be represented as a lottery model. This is a special case of the lottery model in which each agent expresses a weak order over the candidates, similar to the SMT setting [13, 19]. However, the lottery model representation can be exponentially larger than the compact indifference model; for an agent that is indifferent among *n* agents on the other side of the market, there are *n*! possible linearly ordered preferences. The uncertainty models considered in the paper have further been examined in the context of Pareto optimal assignment of items to agents [4–6]. In a subsequent paper, Chen et al. [7] consider additional problems related to the joint probability model.

### Computational Problems

Given a stable marriage setting where agents have uncertain preferences, various natural computational problems arise. Let *stability probability* denote the probability that a matching is stable. We then consider the following two natural problems for each of our uncertainty models.

- StabilityProbability Given a matching and uncertain preferences of the agents, what is the stability probability of the matching?
- MatchingWithHighestStabilityProbability Given uncertain preferences of the agents, compute a matching with the highest stability probability.

We also consider two specific problems that are simpler than StabilityProbability:

- IsStabilityProbabilityNon-Zero For a given matching, is its stability probability non-zero?
- IsStabilityProbabilityOne For a given matching, is its stability probability one?

We additionally consider problems connected to, and more restricted than, MatchingWithHighestStabilityProbability

- ExistsCertainlyStableMatching Does there exist a matching that has stability probability one?
- ExistsPossiblyStableMatching Does there exist a matching that has non-zero stability probability?

Note that ExistsPossiblyStableMatching is straightforward to answer for any of the three uncertainty models we consider here, since there exists a stable matching for each deterministic preference profile that is a possible realization of the uncertain preferences.

**Table 3:** Summary of results

Problems	Lottery model	Compact indifference	Joint probability
StabilityProbability	#P-complete	?	in P
	in P for all three models if 1 side is certain		
IsStabilityProbabilityNon-Zero	NP-complete	in P	in P
IsStabilityProbabilityOne	in P	in P	in P
ExistsPossiblyStableMatching	in P	in P	in P
ExistsCertainlyStableMatching	in P	in P	NP-complete
MatchingWithHighestStabilityProb	?	NP-hard	NP-hard
	in P for all models if 1 side is certain and		
	there is *O*(1) number of uncertain agents		

### Results

Table 3 summarizes our main findings. Note that the complexity of each problem is considered with respect to the input size, and that under the lottery and joint probability models the size of the input could be exponential in *n*, namely $O(n!\cdot 2n)$ for the lottery model and $O((n!)^{2n})$ for the joint probability model, where *n* is the number of agents on either side of the market.

We point out that StabilityProbability is #P-complete for the lottery model even when each agent has at most two possible preferences, but in P if one side has certain preferences. Additionally, we show that IsStabilityProbabilityNon-Zero is in P for the lottery model if each agent has at most two possible preferences. Note that StabilityProbability is open for the compact indifference model when both sides may be uncertain, and we also do not know the complexity of MatchingWithHighestStabilityProbility in the lottery model, except when only a constant number of agents are uncertain on the same side of the market.

## Preliminaries

In the Stable Marriage problem, there are two sets of agents. Let *M* denote a set of *n* men and *W* a set of *n* women. We use the term *agents* when making statements that apply to both men and women, and the term *candidates* to refer to the agents on the opposite side of the market to that of an agent under consideration. Each agent has a linearly ordered preference over the candidates. An agent may be uncertain about his/her linear preference ordering. Let *L* denote the *uncertain preference profile* for all agents.

We say that a given uncertainty model is *independent* if any uncertain preference profile *L* under the model can be written as a product of uncertain preferences $L_a$ for all agents *a*, where all $L_a$’s are independent. Note that the lottery and the compact indifference models are both independent, but the joint probability model is not.

A *matching*$\mu$ is a pairing of men and women such that each man is paired with at most one woman and vice versa; defining a list of (man, woman) pairs (*m*, *w*). We use $\mu (m)$ to denote the woman *w* that is matched to *m* and $\mu (w)$ to denote the match for *w*. Assume that each agent prefers being matched to remaining unmatched. Given linearly ordered preferences, a matching is *stable* if there is no pair (*m*, *w*) not in $\mu$ where *m* prefers *w* to his partner in $\mu$, i.e., $w \succ _m \mu (m)$, and vice versa. If such a pair exists, it constitutes a *blocking pair*; as the pair would prefer to defect and match with each other rather than stay with their partner in $\mu$. A matching $\mu$ is a *complete* matching if all agents are matched in $\mu$. A matching is *certainly stable* if it is stable with probability 1. For an instance $I=(M,W,L)$ and matching $\mu$, let $p(\mu ,I)$ denote the probability of $\mu$ being stable, and let $p_S(I)=max\{p(\mu ,I) \mathbin {: }\mu \text{ is } \text{ a } \text{ matching } \text{ in } I\}$, that is the maximum probability of a matching being stable for *I*.

The following extensions of SM will come in handy in proving our results. The *Stable Marriage Problem with Partially Ordered Lists (SMP)* is an extension of SM in which agents’ preferences are partial orders over the candidates. An instance $I=(M,W,p)$ of SMP is given by a set of *n* men *M*, a set of *n* woman *W*, and the partial preference ordering profile of agents *p* where $p_{a}$ denotes the partial order that represents the preferences of agent *a*. If for a given agent *a* and two candidates *b* and *c* we have that neither *b* is related to *c* nor *c* is related to *b* then *a* cannot compare *b* and *c*. The *Stable Marriage problem with Ties (SMT)* is a special case of SMP in which incomparability is transitive and is interpreted as indifference. Therefore, in SMT each agent partitions the candidates into different ties (equivalence classes), is indifferent between the candidates in the same tie, and has strict preference ordering over the ties. In some practical settings some agents may find some candidates unacceptable and prefer to remain unmatched than to be matched to the unacceptable ones. *SMP with Incomplete Lists (SMPI)* and *SMT with Incomplete lists (SMTI)* captures these scenarios where each agent’s partially ordered list contains only his/her acceptable candidates. Three stability criteria, including the one we have already defined, have been introduced in the literature to capture different degrees of stability for these richer domains. The weakest criterion, that is the one we have already defined, is *(weak) stability*. A matching is *(weakly) stable* in an instance of SMPI if there is no pair $(m,w) \notin \mu$ where *m* (strictly) prefers *w* to his partner in $\mu$ and vice versa. The strongest criterion, referred to by *super-stability*, is closely related to our notion of certain stability. A matching is *super-stable* in an instance of SMPI if there is no pair (*m*, *w*) not in $\mu$ where *m* either prefers *w* to his partner in $\mu$ or finds them incomparable, and vice versa. If such a pair exists, it constitutes a *very weakly blocking pair*. It is easy to observe (see, e.g., [17]) that $\mu$ is super-stable if and only if it is stable w.r.t. all linear extensions of the partially ordered lists.

We define the *certainly preferred* relation $\succ _a^{\text {cert}}$ for agent *a*. We write $b \succ _a^{\text {cert}} c$ if and only if agent *a* prefers *b* over *c* with probability 1. Based on the certainly preferred relation, we can define a dominance relation *D*: $D_{m}(w)=\{w\}\cup \{w'\mathbin {: }w'\succ _m^{\text {cert}} w\}$; $D_{w}(m)=\{m\}\cup \{m'\mathbin {: }m'\succ _w^{\text {cert}} m\}$. Based on the notion of the dominance relation, we present a useful characterization of certainly stable matchings for independent uncertainty models.

### Lemma 1

A matching $\mu$ is certainly stable for an independent uncertainty model if and only if for each pair (*m*, *w*), $\mu (m)\in D_m(w)$ or $\mu (w)\in D_w(m)$.

### Proof

Assume that for each pair (*m*, *w*) we have that $\mu (m)\in D_m(w)$ or $\mu (w)\in D_w(m)$ for a given matching $\mu$. This implies that for each unmatched pair (*m*, *w*) it is the case that $\mu (m) \succ _m^{\text {cert}} w$ or $\mu (w) \succ _w^{\text {cert}} m$ and hence (*m*, *w*) has zero probability of forming a blocking pair. It thus follows that $\mu$ is certainly stable.

Assume that a matching $\mu$ is certainly stable. Then no pair (*m*, *w*) has non-zero probability of forming a blocking pair. This is only possible if the pair (*m*, *w*) is part of the matching or one of *m* and *w* have zero probability of preferring the partner in (*m*, *w*) over their current partner in $\mu$. In either case, $\mu (m)\in D_m(w)$ or $\mu (w)\in D_w(m)$. $\square$

We point out that the certainly preferred relation can be computed in polynomial time for all three models studied in this paper.

## General Results

In this section, we first show that the complexity of all problems that we study is the same for complete and incomplete lists. We then present some general results that apply to multiple uncertainty models. We show that ExistsCertainlyStableMatching can be solved in polynomial time for a class of independent uncertainty models that includes lottery and compact indifference. We then prove that, when the number of uncertain agents is constant and one side of the market is certain, we can solve MatchingWithHighestStabilityProbability efficiently for each of the linear models.

### The Case for Incomplete Lists

The claims in this section explain that our efficient algorithms described for the case of complete lists can be extended to incomplete lists. Additionally, our hardness proofs for incomplete lists can be modified to extend to complete lists. In fact, all our hardness reductions, except Theorem 9, are for complete lists and so they trivially extend to the case of incomplete lists.

In the case of complete lists, we assumed that we have an equal number of men and women and everybody finds all candidates acceptable. When we consider the problem with incomplete lists we mean that the sizes of the two sets are not necessarily the same and not all the candidates are acceptable to all agents. However, we assume that in all realization of the preference profiles the same candidates are acceptable, so we only randomize on the preferences over the acceptable partners.

#### Proposition 1

The computational complexity of StabilityProbability is the same for complete and incomplete lists.

#### Proof

We show that if *I* is an instance of a linear probabilistic model with incomplete lists and $\mu$ is a given matching for *I* then we can, in linear time, construct an extended instance $I'$ with complete lists and a complete matching $\mu '$ for $I'$ such that the stability probability of $\mu$ under *I*, $p(\mu ,I)$, is equal to the stability probability of $\mu '$ under $I'$, $p(\mu ',I')$.

Assume, without loss of generality, that $|M| \ge |W|$. From *I* we create an extended instance $I'$ with sets *M* and $W'$ in the following way. First we ensure that $|M|=|W'|$ by adding enough agents to the woman’s side of the market if necessary. Then we complete the preference lists of each agent by adding the previously unacceptable or nonexistent candidates to the end of her/his list according to a predetermined order, e.g., by the indices of the agents. Suppose now that $\mu$ is a matching in *I* and *X* is the set of matched men in *M*, whilst $\mu (X)=Y$. Let *E* denote the set of acceptable pairs in *I*. We assume that there is no pair $(m,w)\in (M{\setminus } X)\times (W{\setminus } Y)$ belonging to *E*, since in this case this pair would certainly block $\mu$ in *I*, thus $p(\mu , I)=0$ trivially. Let us now extend $\mu$ to another matching $\mu '$ in $I'$ by appending to $\mu$ the unique stable matching for the subinstance restricted to the unmatched agents. Namely, let $\mu _u$ be the stable matching that matches $M{\setminus } X$ to $W'{\setminus } Y$ in such a way that the *k*th pair contains the *k*th man and the *k*th woman from $M{\setminus } X$ and $W'{\setminus } Y$, respectively according to their indices, and let $\mu '=\mu \cup \mu _u$.

We claim that $p(\mu ,I)=p(\mu ',I')$. This is because there is no blocking pair in $(M{\setminus } X)\times (W'{\setminus } Y)$, and any other pair is blocking for some preference profile in *I* if and only if it is blocking for the corresponding preference profile in $I'$. To verify the latter statement, first consider a pair $(m,w)\in X\times Y$. If $(m,w)\in E$ then the partners of *m* and *w* are the same in $\mu$ and $\mu '$ and the ranks of *m* are the same in the corresponding preference profiles of *w*, and vice versa. If $(m,w)\notin E$ then this pair cannot block in any preference profile. Similarly, let us consider a pair (*m*, *w*) when exactly one agent is matched in $\mu$, say, *m*, i.e., $(m,w)\in M\times (W{\setminus } Y)$. If $(m,w)\in E$ then *m* has the same partner in $\mu$ and $\mu '$, whilst *w* is unmatched in $\mu$ and has a previously unacceptable partner in $\mu '$, namely $\mu '(w)$. Whenever this pair blocks for *I* then it also blocks for $I'$, since the rank of *w* is the same in the corresponding preference profiles of *m*, and *w* prefers *m* to $\mu '(w)$ in all profiles. Finally, if $(m,w)\notin E$ then this pair cannot block in any preference profile, as before. $\square$

The above proof implies the following Corollary.

#### Corollary 1

The computational complexity of IsStabilityProbabilityNon-Zero is the same for complete and incomplete lists, and the same holds for IsStabilityProbabilityOne.

#### Proposition 2

The computational complexity of MatchingWithHighestStabilityProbability is the same for complete and incomplete lists.

#### Proof

Suppose that we are given an instance of a linear probabilistic model with incomplete lists *I*, and we consider the extended instance $I'$ with complete preference lists, as described in the proof of Proposition 1. We show that if $\mu '$ is one of the most stable complete matchings in $I'$ then its restriction to *E*, $\mu$, is one of the most stable matching for *I*.

First we note that $p(\mu ,I)\ge p(\mu ',I')$, since any pair in *E* that is blocking for $\mu$ under some preference profile in *I* is also blocking for $\mu '$ under the corresponding extended preference profile in $I'$. Suppose now for a contradiction that there is another matching $\nu$ for *I* such that $p(\nu , I)> p(\mu , I)$. But then, for its natural extension $\nu '$ we have $p(\nu , I)=p(\nu ', I')$ by Proposition 1, contradicting with the maximum stability of $\mu '$. $\square$

The above proof implies the following Corollary.

#### Corollary 2

The computational complexity of ExistsPossiblyStableMatching is the same for complete and incomplete lists, and the same holds for ExistsCertainlyStableMatching.

### An Efficient Algorithm for the Lottery and Compact Indifference Models

As pointed out earlier, certainly stable matchings are closely related to the notion of super-stable matchings [13, 16]. Deciding whether an instance of SMPI admits a super-stable matching or not can be done in polynomial time using algorithm SUPER-SMP in [25]. We next show that for a class of independent uncertainty models that includes the lottery and compact indifference, we can solve ExistsCertainlyStableMatching in polynomial time by a straightforward reduction to the problem of deciding whether an instance of SMP admits a super-stable matching or not.

#### Theorem 1

For any independent uncertainty model in which the certainly preferred relation is transitive and can be computed in polynomial time, ExistsCertainlyStableMatching can be solved in polynomial time.

#### Proof

We prove this by reducing ExistsCertainlyStableMatching to the problem of deciding whether an instance of SMP admits a super-stable matching or not. Let $I=(M,W,L)$ be an instance of ExistsCertainlyStableMatching under an independent uncertainty model in which the certainly preferred relation is transitive and can be computed in polynomial time. We construct in polynomial time an instance $I'=(M,W,p)$ of SMP, where *p* is the agents’ partial preference ordering profile, as follows. The set of men and women are unchanged. To create the partial preference ordering $p_a$ for each agent *a* we do the following. Without loss of generality, assume that *a* is a man *m*. For every pair of women $w_1$ and $w_2$ (i) if $w_1 \succ _m^{\text {cert}} w_2$ then $(w_1,w_2)\in p_m$, denoting that *m* (strictly) prefers $w_1$ to $w_2$ in $I'$, (ii) if $w_2 \succ _m^{\text {cert}} w_1$ then $(w_2,w_1)\in p_m$, denoting that *m* (strictly) prefers $w_2$ to $w_1$ in $I'$. We claim that $I'$ admits a super-stable matching if and only if *I* admits a certainly stable matching. It follows from the definition of super-stability that a matching $\mu$ in $I'$ is super-stable if and only if there is no unmatched pair (*m*, *w*) where $(\mu (m),w) \notin p_m$ and $(\mu (w),m) \notin p_w$.

$(\Leftarrow )$ We first prove that if $I'$ admits a super-stable matching $\mu$ then $\mu$ is certainly stable in *I*. Assume, for a contradiction, that $\mu$ is not certainly stable. It then follows Lemma 1 that $\mu (m)\notin D_m(w)$ and $\mu (w)\notin D_w(m)$, implying that $\mu (m)\not \succ _m^{\text {cert}} w$ and $\mu (w)\not \succ _w^{\text {cert}} m$, and thus $(\mu (m),w) \notin p_m$ and $(\mu (w),m) \notin p_w$. Therefore $\mu$ is not super-stable in $I'$, a contradiction.

$(\Rightarrow )$ Now we prove that if *I* admits a certainly stable matching $\mu$ then $\mu$ is super-stable in $I'$. Assume, for a contradiction, that $\mu$ is not super-stable in $I'$. Therefore there exists an unmatched pair (*m*, *w*) where $(\mu (m),w) \notin p_m$ and $(\mu (w),m) \notin p_w$, implying that $\mu (m)\not \succ _m^{\text {cert}} w$ and $\mu (w)\not \succ _w^{\text {cert}} m$. The latter statement, coupled with the fact that *m* and *w* are not matched together, implies that $\mu (m)\notin D_m(w)$ and $\mu (w)\notin D_w(m)$. Thus, by Lemma 1, $\mu$ is not certainly stable in *I*, a contradiction. $\square$

### An Efficient Algorithm for the Case with a Constant Number of Uncertain Agents

#### Theorem 2

When the number of uncertain agents is constant and one side of the market is certain, then MatchingWithHighestStabilityProbability is polynomial-time solvable for each of the linear models.

#### Proof

Let $I=(M,W,L)$ be an instance of MatchingWithHighestStabilityProbability and assume, without loss of generality, that uncertain agents are all men. Let $X\subseteq M$ be the set of uncertain agents with $|X|=k$ for a constant *k*. We consider all the possible matchings between *X* and *W*; note that their total number is $K=n(n-1)\dots (n-k+1)$. Let $\mu _i$, $i\in \{1\dots K\}$, be such a matching. The main idea of the proof is to show that there exists an extension of $\mu _i$ to $M\cup W$, which we denote by $\mu _i^*$, that has stability probability at least as high as any other extension of $\mu _i$. Furthermore, we can compute $\mu _i^*$ in polynomial time. Therefore, in order to compute a matching that has the highest stability probability, it is enough to generate $\mu _i^*$’s, compute their stability probabilities, compare them and select the one with the highest stability probability. The total number of $\mu _i$’s and hence $\mu _i^*$’s is *K* and hence polynomial in *n*, we can compute each $\mu _i^*$ in polynomial time (as we will see later in this proof) and computing the stability probability of a given $\mu _i^*$ can be done in polynomial time since all uncertain agents are on one side of the market (see Theorem 3 in Section 4, Theorem 8 in Section 5 and Theorem 10 in Section 6).

Take a matching $\mu _i$ between sets *X* and *W*. Let $Y=\mu _i(X)$ (i.e., the partners of *X* in *W*) and let $M'=M{\setminus } X$ and $W'=W{\setminus } Y$. Recall that all agents in $M'\cup W$ are certain. First, we compute the man-optimal matching $\mu _i^M$ for the sub-instance $I'$ on $M'\cup W'$, that can be done efficiently by the Gale-Shapley algorithm [11]. Consider the matching $\mu '_i = \mu _i \cup \mu _i^M$ in *I*. If $\mu '_i$ admits a blocking pair $(m',w)$ involving some (certain) agents $m'\in M'$ and $w\in Y$ (that we will refer to as a BP_Type1 blocking pair) then we can conclude that any extension of $\mu _i$ to a matching in *I* will have zero probability of being stable. This is because any extension of $\mu _i$ that has a positive probability of being stable in *I* must also be stable for the sub-instance $I'$. If $(m',w)$ is a blocking pair for $\mu '_i$ then it also blocks any other extension of $\mu _i$ in *I*, since in all extensions *w* has the same partner and $m'$ cannot have a better partner than in $\mu _i^M$. Therefore we can exclude the extensions of $\mu _i$ from further consideration in this case.

Suppose now that $\mu '_i = \mu _i \cup \mu _i^M$ admits no BP_Type1 blocking pair. We truncate the preference lists of men in $M'$ in the following way. For each man $m'\in M'$ we remove from his preference list all the women $w'\in W'$ that $m'$ prefers less than some woman $w \in Y$ who prefers $m'$ to her partner in $\mu _i$. That is, we remove $w'$ from the list of $m'$ if there exists $w\in Y$ such that $w\succ _{m'}w'$ and $m'\succ _{w} \mu _i(w)$. To do this, it is enough to identify the first (i.e. the highest ranked) woman *w* in the preference list of $m'$ such that $w\in Y$ and $m'\succ _{w} \mu _i(w)$, and then remove from the preference list of $m'$ all women $w'\in W'$ that appear after *w*. Let us denote the sub-instance for $M'\cup W'$ with the truncated lists as $I_i^r$. Now we compute the woman-optimal matching $\mu _i^W$ in $I_i^r$. Let $\mu _i^*=\mu _i\cup \mu _i^W$. We claim that $\mu _i^*$ is a complete matching in *I* and is stable for the certain agents; that is, no blocking pair (*m*, *w*) exists where both *m* and *w* are certain agents. To see this, first note that since $\mu '_i$ admits no BP_Type1 blocking pair hence $\mu _i^M(m')$ remains in the truncated preference list of all $m'\in M'$. Therefore, the man-optimal matching for $I_i^r$ is the same as the man-optimal matching for $I'$, $\mu _i^M$, implying that a complete stable matching exists in $I_i^r$ and that each $m' \in M'$ is either matched to the same woman in both $\mu _i^M$ and $\mu _i^W$ or prefers $\mu _i^M(m')$ to $\mu _i^W(m')$. As all stable matchings in $I_i^r$ are of the same size (by the Rural-Hospital Theorem, see e.g. [26]), hence all agent in $I_i^r$ are matched in $\mu _i^W$ and therefore $\mu _i^*$ is a complete matching for *I*. Now assume, for a contradiction, that $\mu _i^*$ is not stable for the certain agents and hence admits a blocking pair $(m^*, w^*)$ where $m^* \in M'$ and $w^*\in W$. Note that $w^* \notin W'$ as otherwise $(m^*, w^*)$ blocks $\mu _i^W$ in $I_i^r$, a contradiction. Therefore, $w^* \in Y$. But then we have that $w^* \in Y$ prefers $m^* \in M'$ to $\mu _i(w^*)$ and also that $m^*$ prefers $w^*$ to $\mu _i^W(m^*)$, implying that by the construction of the truncated preference lists of men in $M'$, woman $\mu _i^W(m^*)$ must have been removed from $m^*$’s preference list, a contradiction. Therefore $\mu _i^*$ is a complete matching in *I* and is stable for certain agents.

Finally, we show that for any matching $\mu _i^+$ that is an extension of $\mu _i$ to *I*, the stability probability of $\mu _i^+$ is less than, or equal to, the stability probability of $\mu _i^*$. If $\mu _i^+$ is not stable for the certain agents then $\mu _i^+$ has zero probability of being stable, thus the statement holds. Otherwise, $\mu _i^+$ is stable for the certain agents and hence for any pair (*m*, *w*) that has a positive probability of being a blocking pair it must be that $m\in X$ and $w\in W'$. Moreover, the projection of $\mu _i^+$ onto $I_i^r$ must be stable in $I_i^r$, and as each woman $w\in W'$ is matched in $\mu _i^*$ to her optimal stable partner under $I_i^r$ thus *w* either prefers her partner in $\mu _i^*$ to her partner in $\mu _i^+$, or is matched to the same partner under both matchings. Therefore, for each deterministic preference profile that has a positive probability of realization under *I*, if a pair (*m*, *w*) blocks $\mu _i^*$ then it must also block $\mu _i^+$, implying that if $\mu _i^+$ is stable under the given preference profile then so is $\mu _i^*$, and thus our statement follows. $\square$

## Lottery Model

In this section, we turn to the lottery model. Recall that in the lottery model, each agent reports a probability distribution over a set of preference orders. We show that even with small supports, computing StabilityProbability is still a computationally hard problem. However, other questions become more tractable with small supports or one side having certain preferences.

### Theorem 3

For the lottery model, if one side has certain preferences, StabilityProbability is polynomial-time solvable.

### Proof

Without loss of generality, assume that men have certain preferences so each man *m* has a deterministic preference relation $\succ _m$. We present a polynomial-time computable formula for the probability of $\mu$ being stable. For a woman *w*, we denote her set of possible preferences lists by $P_w=\{\succ _w^{P_w^1}, \ldots , \succ _w^{P_w^{k_w}}\}$ with each preference list $\succ _w^{P_w^i}$ having corresponding probability $p_w^i$. Let $q_w$ be the probability that woman *w* will not form a blocking pair. The term $q_w$ is by definition as follows.

$$\begin{aligned} q_w=\sum _{\succ _w^{P_w^j}\in P_w\mathbin {: }~ \not \exists ~m~\in M \text { for which } w~\mathrel {\succ _m} ~\mu (m)\text { and } m~\mathrel {\succ _w^{P_w^j}}~\mu (w)}p_w^j \end{aligned}$$

So for each $w\in W$, $q_w$ can be computed in time $O(k_wn)$ by scanning over the $k_w$ preference lists of woman *w* and checking in which of them it is not the case that some *m* prefers *w* over $\mu (m)$ and *w* prefers *m* over $\mu (w)$. The probability that $\mu$ is stable is equal to the probability that no woman is in a blocking pair:

$$\begin{aligned} p({\mu \text { is stable})} = \prod _{w\in W} q_w. \end{aligned}$$

$$\square $$

### Theorem 4

For the lottery model, IsStabilityProbabilityOne can be solved in polynomial time.

### Proof

The problem is equivalent to checking whether the given matching $\mu$ has non-zero probability of *not* being stable, i.e., we need only find a single possible blocking pair. This can be checked as follows: for each pair (*m*, *w*) of agents that are not matched to each other, we check whether either member of this pair can form a blocking pair with a pair in $\mu$ with non-zero probability. To do this, we need to check whether *m* prefers *w* in some possible preference over $\mu (m)$ and whether *w* prefers *m* in some possible preference over $\mu (w)$. As this only involves checking $n^2 - n$ possible pairs, each of which can be checked in linear time, this can be done in polynomial time. $\square$

### Theorem 5

For the lottery model, IsStabilityProbabilityNon-Zero is polynomial-time solvable when each agent has at most two possible preference orderings.

### Proof

The problem is to decide whether there is some preference ordering for each agent (among the ones in their lottery) such that the given matching is stable. If each agent has at most two possible preference orderings in their lottery, we can reduce the problem to an instance $\varphi$ of 2SAT [12], as follows. 2SAT is the problem of deciding whether a given propositional formula in 2CNF is satisfiable. A propositional formula $\varphi$ is in 2CNF if it is the conjunction of clauses $(l_1 \vee l_2)$ of size 2, where $l_1$ and $l_2$ are propositional literals (propositional variables *x* or their negation $\lnot x$).

Let $\{ a_1,\dotsc ,a_n \}$ and $\{ b_1,\dotsc ,b_n \}$ be the two sets of agents. Moreover, for each agent *c* and each $i \in \{ 1,2 \}$, let $\text {pref}(c,i)$ denote the *i*-th preference in the lottery for agent *c*.

We introduce a propositional variable for each preference $\text {pref}(c,i)$, which we also call $\text {pref}(c,i)$. Intuitively, these variables indicate which preference for the agents we choose to make the matching stable.

For each agent *c*, we add the following clauses to $\varphi$, to ensure that for each agent *c* there is exactly one preference that is selected: $(\text {pref}(c,1) \vee \text {pref}(c,2)) \quad \wedge \quad (\lnot \text {pref}(c,1) \vee \lnot \text {pref}(c,2)).$

Then, we add clauses to ensure that the selected matching is stable. For each agent *c* and each $i \in \{ 1,2 \}$, let $B_{c,i}$ be the set of preferences $\text {pref}(c',i')$—for $c' \ne c$ and $i' \in \{ 1,2 \}$—such that $\text {pref}(c,i)$ and $\text {pref}(c',i')$ together lead to the given matching being unstable (with $(c,c')$ being a blocking pair). Then, for each *c*, *i*, we add the following clauses: $(\lnot \text {pref}(c,i) \vee \lnot \text {pref}(c',i')) \quad \text {for each}~\text {pref}(c',i') \in B_{c,i}$.

The given matching is then stable if and only if $\varphi$ is satisfiable. Since $\varphi$ is a 2CNF formula, this can be decided in linear time [1]. $\square$

### Theorem 6

For the lottery model, StabilityProbability is #P-complete, even when each agent has at most two possible preferences.

### Proof

We show how to count the number of satisfying assignments for a 2CNF formula using the problem StabilityProbability for the lottery model where each agent has two possible preferences. Since this problem is #P-hard, we get #P-hardness also for StabilityProbability [23].

Let $\varphi$ be a 2CNF formula over the variables $x_1,\dotsc ,x_n$. We firstly transform $\varphi$ to a 2CNF formula $\varphi '$ over the variables $x_1,\dotsc ,x_{2n},y_1,\dotsc ,y_{2n}$ that has exactly the same number of satisfying assignments, and that satisfies the property that each clause contains one variable among $x_1,\dotsc ,x_{2n}$ and one variable among $y_1,\dotsc ,y_{2n}$. We do so as follows. Firstly, for each $1 \le i \le n$, we add the clauses $(\lnot x_i \vee y_{i})$, $(\lnot y_{i} \vee x_{n+i})$,  $(\lnot x_{n+i} \vee y_{n+i})$ and $(\lnot y_{n+i} \vee x_{i})$, ensuring that in each satisfying assignment the variables $x_i$, $x_{n+i}$, $y_i$ and $y_{n+i}$ get assigned the same truth value. Then, for each clause of $\varphi$, we replace one occurrence of a variable among $x_1,\dotsc ,x_n$ by a corresponding variable among $y_1,\dotsc ,y_{2n}$—that is, we replace $x_i$ either by $y_i$ or by $y_{n+i}$—and we add the resulting clause to $\varphi '$. For example, if $\varphi$ contains the clause $(x_1 \vee \lnot x_3)$, we could add the clause $(x_1 \vee \lnot y_3)$ to $\varphi '$. It is readily verified that $\varphi '$ has the same number of satisfying assignments as $\varphi$.

Without loss of generality, we may assume that for each pair $x_i,x_j$ of variables among $x_1,\dotsc ,x_n$, the original formula $\varphi$ contains at most two distinct clauses that contain both $x_i$ and $x_j$. If $\varphi$ were to contain three or more distinct clauses that contain both $x_i$ and $x_j$, we know that at least one of the literals $x_i,\lnot x_i,x_j,\lnot x_j$ would be entailed by these clauses, and we could instantiate these entailed literals and simplify $\varphi$ accordingly. For example, if $\varphi$ contains the clauses $(x_1 \vee x_2)$, $(x_1 \vee \lnot x_2)$ and $(\lnot x_1 \vee \lnot x_2)$, we can simplify $\varphi$ by instantiating the entailed literals $x_1$ and $\lnot x_2$. As a result, we know that we can construct $\varphi '$ in such a way that for any two variables of $\varphi '$, there is at most one clause of $\varphi '$ that contains both of these variables. For instance, if $\varphi$ contains the clauses $(x_1 \vee x_2)$ and $(\lnot x_1 \vee \lnot x_2)$, we can construct $\varphi '$ in such a way that it contains the clauses $(x_1 \vee y_2)$ and $(\lnot y_1 \vee \lnot x_2)$.

We now construct an instance of StabilityProbability. The sets of agents that we consider are $\{ x_1,\dotsc ,x_{2n}, a_1,\dotsc ,a_{2n} \}$ and $\{ y_1,\dotsc ,y_{2n},b_1,\dotsc ,b_{2n} \}$. The matching that we consider matches $x_i$ to $b_i$ and matches $y_i$ to $a_i$, for each $1 \le i \le 2n$ as shown below.
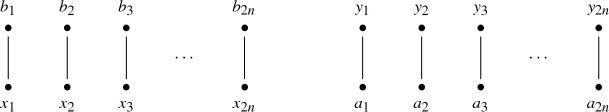


Each agent $b_i$ has only a single possible preference, namely one where they prefer $x_i$ over all other agents. Similarly, each agent $a_i$ has a single possible preference where they prefer $y_i$ over all other agents. In other words, the agents $a_i$ and $b_i$ are perfectly happy with the given matching.

The agents $x_i$ and $y_i$ each have two possible preferences, that are each chosen with probability $\frac{1}{2}$. These two possible preferences are associated with setting these variables to true or false, respectively. We describe how these preferences are constructed for the agents $x_i$. The construction for the preferences of the agents $y_i$ is then entirely analogous.

Take an arbitrary agent $x_i$. We show how to construct the two possible preferences for agent $x_i$, which we denote by $p_{x_i}$ and $p_{\lnot x_i}$. Both of these possible preferences are based on the following partial ranking: $b_1> b_2> \cdots > b_{2n},$ and we add some of the agents among $y_1,\ldots ,y_{2n}$ to the top of this partial ranking, and the remaining agents to the bottom of this partial ranking.

To the ranking $p_{x_i}$ we add exactly those agents $y_j$ to the top where $\varphi '$ contains a clause $(\lnot x_i \vee y_j)$ or a clause $(\lnot x_i \vee \lnot y_j)$. All remaining agents we add to the bottom. Similarly, to the ranking $p_{\lnot x_i}$ we add exactly those agents $y_j$ to the top where $\varphi '$ contains a clause $(x_i \vee y_j)$ or a clause $(x_i \vee \lnot y_j)$. The rankings $p_{y_j}$ and $p_{\lnot y_j}$, for the agents $y_j$, are constructed entirely similarly.

Consider a truth assignment $\alpha : \{ x_1,\dotsc ,x_{2n}, y_1,\dotsc ,y_{2n} \} \rightarrow \{ 0,1 \}$, and consider the corresponding choice of preferences for the agents $x_1,\dotsc ,x_{2n},y_1,\dotsc ,y_{2n}$, where for each agent $x_i$ the preference $p_{x_i}$ is chosen if and only if $\alpha (x_i) = 1$, and for each agent $y_j$ the preference $p_{y_j}$ is chosen if and only if $\alpha (y_j) = 1$. We show that $\alpha$ satisfies $\varphi '$ if and only if the corresponding choice of preferences leads to the matching being stable. The only blocking pairs that can arise are between an agent $x_i$ and an agent $y_j$. Take an arbitrary such pair $(x_i,y_j)$. We show that this is a blocking pair if and only if $\alpha$ falsifies at least one clause containing both variables $x_i$ and $y_j$. We know that $\varphi '$ contains exactly one clause containing $x_i$ and $y_j$. We deal with the case where $\varphi '$ contains the clause $(x_i \vee y_j)$—the other possibilities are entirely analogous. By construction of $p_{x_i},p_{\lnot x_i},p_{y_j}$, and $p_{\lnot y_j}$, we get that $(x_i,y_j)$ is a blocking pair if and only if both $p_{\lnot x_i}$ and $p_{\lnot y_j}$ are chosen. This is exactly the case where the clause $(x_i \vee y_j)$ is falsified. Thus, we can conclude that $\alpha$ falsifies $\varphi '$ if and only if there is a blocking pair, and thus that $\alpha$ satisfies $\varphi '$ if and only if the corresponding choice of preferences leads to the matching being stable

Since each combination of preferences is equally likely to occur, and there are $2^{4n}$ many combinations of preferences, the probability that the given matching is stable is exactly $q = \frac{s}{2^{4n}}$, where *s* is the number of satisfying truth assignments for $\varphi$. Therefore, given *q*, *s* can be obtained by computing $s = q 2^{4n}$. $\square$

If the agents are allowed to have more than two possible preferences as we have assumed in the last two Theorems, then even the following problem is NP-complete. The statement can be proved via a reduction from Exact Cover by 3-Sets (X3C).

### Theorem 7

For the lottery model, IsStabilityProbabilityNon-Zero is NP-complete.

### Proof

The problem is in NP, since we only need to provide one profile that occurs with non-zero probability for which the given matching is stable. We show NP-hardness by giving a reduction from Exact Cover by 3-Sets (X3C). Let (*X*, *C*) be an instance of X3C, where $|X| = 3n$ for some *n*, and $C = \{ c_1,\dotsc ,c_m \}$ is a collection of sets $c_i \subseteq X$, each of size 3. Moreover, let $c_i = \{ x_{\ell _{i,1}}, x_{\ell _{i,2}}, x_{\ell _{i,3}} \}$, for each $1 \le i \le m$. The problem is to decide whether there is a subset $C'\subseteq C$ of size exactly *n* such that $\bigcup C' = X$.

We construct an instance of our problem as follows. We let $\{ a_1,\dotsc ,a_{n}, {}a'_1,\dotsc ,a'_{3n} \}$ and $\{ b_1,\dotsc ,b_{n},b'_1,\dotsc ,b'_{3n} \}$ be the two sets of agents, we match $a_i$ to $b_i$—for each $1 \le i \le n$— and we match $a'_j$ to $b'_j$—for each $1 \le j \le 3n$. This is depicted graphically below.
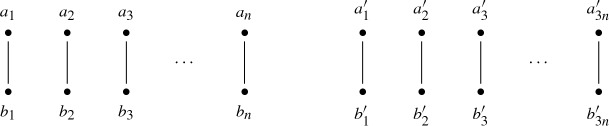


Each agent $a_i$ prefers their matching to $b_i$ over any other possible match, i.e., agent $a_i$ has one preference, where $b_i$ is ranked first, and the rest of the agents appear in some fixed order after $b_i$.

Similarly, each agent $b'_j$ prefers their matching to $a'_j$ over any other possible match. That is, agent $b'_j$ has one preference ordering in which $a'_j$ is ranked first and the rest of the agents appear in some fixed order after $a'_j$.

Then, for each agent $b_i$, we add the following |*C*| possible preferences to the lottery:

$$\begin{aligned} P_{i,1}&: \quad a'_{\ell _{1,1}}> a'_{\ell _{1,2}}> a'_{\ell _{1,3}}> a_i> \cdots \\ P_{i,2}&: \quad a'_{\ell _{2,1}}> a'_{\ell _{2,2}}> a'_{\ell _{2,3}}> a_i> \cdots \\& \vdots \\ P_{i,m}&: \quad a'_{\ell _{m,1}}> a'_{\ell _{m,2}}> a'_{\ell _{m,3}}> a_i > \cdots \end{aligned}$$

where in each preference the remaining agents appear in any (fixed) order after $a_i$. In other words, $b_i$ prefers three agents $a'_j$ to their current match, and these three form some set $c \in C$.

Finally, for each agent $a'_j$, we add the following *n* possible preferences to the lottery:

$$\begin{aligned} \begin{array}{r r} P'_{j,1} : \quad &{} b_2> \cdots> b_n> b'_j> b_1> b'_1> \cdots> b'_{j-1}> b'_{j+1}> \cdots> b'_{3n} \\ P'_{j,2} : \quad &{} b_1> b_3> \cdots> b_n> b'_j> b_2> b'_1> \cdots> b'_{j-1}> b'_{j+1}> \cdots> b'_{3n}\\ P'_{j,3} : \quad &{} b_1> b_2> b_4> \cdots> b_n> b'_j> b_3> b'_1> \cdots> b'_{j-1}> b'_{j+1}> \cdots> b'_{3n} \\ \vdots \quad \ &{} \vdots \qquad \quad \ \\ P'_{j,n} : \quad &{} b_1> \cdots> b_{n-1}> b'_j> b_n> b'_1> \cdots> b'_{j-1}> b'_{j+1}> \cdots > b'_{3n} \end{array} \end{aligned}$$

That is, each agent $a'_j$ prefers each of the agents $b_1,\dotsc ,b_n$, except one, to their current match (and they never prefer any of the agents $b'_{j'}$ for $j' \ne j$ over their current match).

We can show that there is a choice of preferences for the agents that makes this matching stable if and only if $(X,C) \in \text {X3C}$.

$(\Rightarrow )$ Firstly, suppose that there is a choice of preferences for the agents that makes this matching stable. That is, for each agent $b_i$ there is some preference ordering $P_{i,\ell _i}$, and for each agent $a'_j$ there is some preference ordering $P'_{j,k_j}$, such that these orderings (together with the fixed preference orderings for the agents $a_i$ and $b'_j$) make this matching stable. Now, consider the set $C' = \{ c_{\ell } : i \in [n], \ell = \ell _i \}$. We show that $\bigcup C' = X$. To derive a contradiction, suppose that this is not the case, that is, suppose that $\bigcup C' \ne X$. Then, since $|C'| = n$, $|X| = 3n$ and for each $c \in C'$ it holds that $|c| = 3$, we know that there must be some $c_{\ell },c_{\ell '} \in C'$ such that $c_{\ell } \cap c_{\ell '} \ne \emptyset$. Say that $x_j \in c_{\ell } \cap c_{\ell '}$. Therefore, there must be some $i,i' \in [n]$ such that both $b_i$ and $b_{i'}$ prefer $a'_{j}$ over their current match. On the other hand, $a'_{j}$ will prefer either $b_i$ or $b_{i'}$ over their current match. Therefore, either $b_i$ and $a'_{j}$ or $b_{i'}$ and $a'_{j}$ will form a blocking pair. Thus, the matching is not stable. From this we can conclude that $\bigcup C' = X$.

$(\Leftarrow )$ Conversely, suppose that there exists some $C' \subseteq C$ of size exactly *n* such that $\bigcup C' = X$. Let $C' = \{ c_{\ell _1},\dotsc ,c_{\ell _n} \}$. Now, for each agent $b_i$ we pick some preference ordering, and for each agent $a'_j$ we pick some preference ordering, such that these orderings (together with the fixed preference orderings for the agents $a_i$ and $b'_j$) make the matching stable. For each agent $b_i$, we pick the preference ordering $P_{i,\ell _i}$, and for each agent $a'_j$ we pick the preference ordering $P'_{j,k_j}$, where $k_j \in [n]$ is the unique value such that $x_{j} \in c_{\ell _{k_j}}$. It is straightforward to verify that these preferences make the matching stable. $\square$

Given that StabilityProbability is computationally hard even in restricted settings, one may immediately wonder about approximation algorithms. However, we can state the following two corollaries, the first from Theorem 7 and the second from [29, Proposition 8] and Theorem 7, which show that even approximation remains intractable.

### Corollary 3

For the lottery model, unless P=NP, there exists no polynomial-time constant-factor approximation algorithm StabilityProbability of a given matching.

### Corollary 4

For the lottery model, unless NP=RP, there is no FPRAS for StabilityProbability.

## Compact Indifference Model

In the compact indifference model we are given an instance of SMT and each linear order over candidates (each possible preference ordering) is achieved by breaking ties independently at random with uniform probabilities. It is easy to show that IsStabilityProbablityNonZero, IsStabilityProbablityOne, and ExistsCertainlyStableMatching are all in P.

### Proposition 3

For the compact indifference model, IsStabilityProbabilityNonZero is in P.

### Proof

This is equivalent to checking whether a given matching $\mu$ is weakly stable in the given SMTI instance, which is polynomial-time solvable. To check this we only have to look for a blocking pair, which can be done in polynomial time: take every possible pair (*m*, *w*) who are not matched together and check whether they both (strictly) prefer each other to their current partner. $\square$

### Proposition 4

For the compact indifference model, IsStabilityProbabilityOne is in P.

### Proof

This is equivalent to checking whether a given matching $\mu$ is super stable in the given SMTI instance, which is polynomial-time solvable. To check this we only have to look for a very weakly blocking pair, which can be done in polynomial time: take every possible pair (*m*, *w*) who are not matched together and check whether they each either prefers the other to his or her current partner or finds them incomparable.

$$\square $$

### Proposition 5

For the compact indifference model, ExistsCertainlyStableMatching is in P.

### Proof

Deciding whether there is a matching that is stable with probability one is equivalent to deciding whether there is a matching that is stable w.r.t. all refinements, i.e., a super-stable matching. Given an instance of SMTI one can decide in polynomial time whether it admits a super-stable matching [20]. $\square$

The complexity of computing the stability probability of a given matching remains open under the compact indifference model, but this problem can be shown to be in P if one side has certain preferences.

### Theorem 8

In the compact indifference model, if one side has certain preferences, StabilityProbability is polynomial-time solvable.

### Proof

Assume, without loss of generality, that men have certain preferences. The following procedure gives us the stability probability of any given matching $\mu$. (1) For each uncertain woman *w* identify those men with whom she can potentially form a blocking pair. That is, those *m* such that $w~{\succ }_{m}~\mu (m)$ and *w* is indifferent between *m* and her partner in $\mu$. Assume there are *k* such men. The probability of *w* not forming a blocking pair with any men is then the probability that her current match ranks first among these $k+1$ men, which is $\frac{1}{k+1}$. (2) Multiply the probabilities from step 1. $\square$

We next show that MatchingWithHighestStabilityProbability is NP-hard. For an instance *I* of SMT and matching $\mu$, let $p(\mu ,I)$ denote the probability of $\mu$ being stable, and let $p_S(I)=max\{p(\mu ,I)\, |\, \mu \text{ is } \text{ a } \text{ matching } \text{ in } I\}$, that is the maximum probability of a matching being stable. A matching $\mu$ is said to be weakly stable if there exists a tie-breaking rule where $\mu$ is stable. Therefore a matching $\mu$ has positive probability of being stable if and only if it is weakly stable. Furthermore, if the number of possible tie-breaking is *N* then any weakly stable matching has a probability of being stable at least $\frac{1}{N}$.

An extreme case occurs if we have one woman only with *n* men, where the woman is indifferent between all men. In this case any matching (pair) has a $\frac{1}{n}$ probability of being stable. An even more unfortunate scenario is when we have *n* men and *n* women, each women is indifferent between all men, and each man ranks the women in a strict order in the same way, e.g., in the order of their indices. In this case, the probability that the first woman picks her best partner, and thus does not block any matching is $\frac{1}{n}$. Suppose that the first woman picked her best partner, the probability that the second woman also picks her best partner from the remaining $n-1$ men is $\frac{1}{n-1}$, and so on. Therefore, the probability that an arbitrary complete matching is stable is $\frac{1}{n\cdot (n-1)\cdot \dots \cdot 2}=\frac{1}{n!}$.

### Theorem 9

For the compact indifference model, MatchingWithHighestStabilityProbability is NP-hard, even if only one side of the market has uncertain agents.

### Proof

For an instance *I* of SMTI, let *opt*(*I*) denote the maximum size of a weakly stable matching in *I*. [14] showed (in the proof of Corollary 3.4) that given an instance *I* of SMTI of size *n*, where only one side of the market has agents with indifferences and each of these agents has a single tie of size two, and any arbitrary small positive $\epsilon$, it is NP-hard to distinguish between the following two cases: (1) $opt(I) \ge \frac{21-\epsilon }{27}n$ and (2) $opt(I) < \frac{19+\epsilon }{27}n$.

When choosing $\epsilon$ so that $0<\epsilon <\frac{1}{2}$ we can simplify the above cases to (1) $opt(I) > \frac{41}{54}n$, since $opt(I) \ge \frac{21-\epsilon }{27}n > \frac{41}{54}n$ and (2) $opt(I) < \frac{39}{54}n$, since $opt(I)< \frac{19+\epsilon }{27}n < \frac{39}{54}n$.

Therefore, the number of agents left unmatched on either side of the market is less than $\frac{13}{54}n$ in the first case and more than $\frac{15}{54}n$ in the second case. Let us now extend instance *I* to a larger instance of SMTI $I'$ as follows. Besides the *n* men $M=\{m_1, \dots , m_n\}$ and *n* women $W=\{w_1, \dots ,w_n\}$, we introduce $\frac{13}{54}n$ men $X=\{x_1,\dots x_k\}$ and another $\frac{n}{27}$ men $Y=\{y_1,\dots y_l\}$ and $\frac{n}{27}$ women $Z=\{z_1,\dots z_l\}$. Furthermore, for each $y_j\in Y$, we introduce *n* men $Y^j=\{y^j_1,\dots , y^j_n\}$. We create the preferences of $I'$ as follows. The preferences of men *M* remain the same. For each woman $w\in W$ we append the men *X* and then *Y* at the end of her list in the order of their indices. Each man $x_i\in X$ has only all the women *W* in his list in the order of their indices. Furthermore, each $y_j\in Y$ has all the women *W* first in his preference list in the order or their indices and then $z_j$. Let each $z_j\in Z$ has $y_j$ as first choice and then all the men $Y^j$ in one tie of size *n*. Each man in $Y^j$ has only $z_j$ in his list. We will show that in case one $p_S(I')\ge \frac{1}{2^n}$, whilst in case two $p_S\le (\frac{1}{n})^{\frac{n}{27}}$. Therefore, for $n>2^{27}$, it is NP-hard to decide which of the two separate intervals contains the value $p_S(I')$.

To show the above statement, suppose first that we have the first case, so $opt(I) > \frac{41}{54}n$ and therefore less than $\frac{13}{54}n$ women are left unmatched in a maximum size weakly stable matching $\mu$ for *I*, denoted by $W_u\subset W$. We extend $\mu$ to $\mu '$ for $I'$ as follows. We assign all the women in $W_u$ to men in *X* in the unique stable way, namely we pair them in a mutually increasing order of their indices. Since $|X|>|W_u|$, we now matched all women in *W*, and left some men in *X* unmatched in $\mu '$. We complete the matching by assigning $y_j$ to $z_j$ for each $j=1,\dots ,n$ and leaving all of the men in $Y^j$ for all *j* unmatched. We shall see that no matter how we break the ties in $I'$, blocking pair can appear between the original *I* agents only, and therefore the probability of $\mu '$ being stable in $I'$ is the same as the probability of $\mu$ being stable in *I*. Since we have at most *n* ties in *I*, each of length two, the number of different tie-breakings is at most $2^n$, out of which at least one is stable. Therefore $p(\mu ,I')=p(\mu ,I)\ge \frac{1}{2^n}$.

In the second case, $opt(I) < \frac{39}{54}n$ and therefore more than $\frac{15}{54}n$ women are left unmatched in any weakly stable matching $\mu$ for *I*. Let $\mu '$ be one of the most stable matchings in $I'$. First we have to note that the restriction of $\mu '$ to *I* must be weakly stable in *I*, since otherwise $p(\mu ',I')=0$. Let $W_u$ denote the set of women that are not matched to any man from *M* in $\mu '$. According to our assumption $|W_u|>\frac{15}{54}n$, whilst $|X|+|Y|=\frac{15}{54}n$, therefore in order to avoid a certain blocking pair between $W_u$ and $X\cup Y$ we shall match all the men in $X\cup Y$ to women in $W_u$ in the only stable way (in the order of indices, where men in *X* are coming before men in *Y*), leaving some women in $W_u$ unmatched in $\mu '$. However, in this case no agent $z_j\in Z$ can be matched to $y_j$, and therefore, even if there was no potential blocking pair between agents of *I*, the probability that $z_j$ is matched the best partner from $Y^j$ is $\frac{1}{n}$ independently for each $z_j\in Z$. Therefore the probability of $\mu '$ being stable is at most $(\frac{1}{n})^{\frac{n}{27}}$, which completes the proof of the first statement.

Regarding the NP-hardness of finding one of the most stable matchings, we shall prove that we can decide between the two cases according to the number of unmatched women in *W* in the restriction of $\mu '$ to *I*, where $\mu '$ is one of the most stable matchings in $I'$. To see this, let $W_u$ denote again the set of women that are not matched to any man in *M* under $\mu '$. In the first case, when $opt(I) > \frac{41}{54}n$, it must be the case that $|W_u|<\frac{15}{54}n$, since otherwise $p(\mu ',I)$ would be less than $(\frac{1}{n})^{\frac{n}{27}}$ and could not achieve $\frac{1}{2^n}$, that is the minimum value for $p_S(I')$, as shown in the above argument. Whilst, in the second case $|W_u|>\frac{15}{54}n$ must hold, since $opt(I) < \frac{39}{54}n$ was assumed. $\square$

## Joint Probability Model

In this section, we examine problems concerning the joint probability model. Recall that the input now consists of a set of preference profiles, and a probability distribution over these preference profiles. Our first result is that the stability probability of a matching can be computed in polynomial time in the size of the input.

### Theorem 10

For the joint probability model, StabilityProbability can be solved in polynomial time.

### Proof

The probability that a given matching is stable is equivalent to the sum of the probability weights of the preference profiles for which the matching is stable. This can be checked as follows. We check for which preference profiles the given matching is stable. For one profile, this can be checked in $O(n^2)$ time by checking for each (man, woman) pair, whether it is a blocking pair. Then we add up the probabilities of those profiles for which the matching is stable. The sum of the probabilities is the probability that the matching is stable. $\square$

### Corollary 5

For the joint probability model, IsStabilityProbabilityNon-Zero and IsStabilityProbabilityOne can be solved in polynomial time.

For the joint probability model, the problem ExistsCertainlyStableMatching is equivalent to checking whether the intersection of the sets of stable matchings of the different preference profiles is empty.

### Theorem 11

For the joint probability model, ExistsCertainlyStableMatching is NP-complete.

### Proof

The problem is in NP, since computing StabilityProbability can be done in polynomial time by Theorem 10 , and so a matching that is stable with probability 1 is a certificate. The NP-hardness proof is by reduction from the NP-complete 3-Coloring problem. The input for 3-Coloring is a graph $G=(V,E)$, and the question is whether there exists a coloring of the vertex set with 3 colors, $\chi : V \rightarrow \{ 1,2,3 \}$, such that no two adjacent vertices receive the same color. Such a coloring is called a *proper* coloring. Let $G = (V,E)$ be a graph specifying an instance of 3-Coloring, where $V = \{ v_1,\dotsc ,v_n \}$. We construct an instance *I* of ExistsCertainlyStableMatching in the joint probability model.

For each vertex $v_i \in V$, we introduce three men $m_{i,1}, m_{i,2}, m_{i,3}$ and three women $w_{i,1}, w_{i,2}, w_{i,3}$. We will add a sequence of preference profiles that have non-zero probability. The exact probability of the preference profiles is unimportant, so we may assume that there is a uniform probability associated with the preference profiles. The first preference profile $P_0$ will ensure that every certainly stable matching matches each $m_{i,j}$ to some $w_{i,j'}$ and each $w_{i,j}$ to some  $m_{i,j''}$, where $j,j',j'' \in \{1,2,3\}$. Moreover, it ensures that for each  $i \in \{1,\dots ,n\}$, exactly one of three matchings between the men $m_{i,j}$ and the women $w_{i,j}$ must be used:

$$\begin{aligned} \begin{array}{r l} \text {(1)} &{} m_{i,1}\text { is matched to}~w_{i,1}\text {, }m_{i,2}\text { is matched to}~w_{i,2}\text {, and }m_{i,3}\text { is matched to}~w_{i,3}; \\ \text {(2)} &{} m_{i,1}\text { is matched to}~w_{i,2}\text {, }m_{i,2}\text { is matched to}~w_{i,3}\text {, and }m_{i,3}\text { is matched to}~w_{i,1}\text {; or} \\ \text {(3)} &{} m_{i,1}\text { is matched to}~w_{i,3}\text {, }m_{i,2}\text { is matched to}~w_{i,1}\text {, and }m_{i,3}\text { is matched to}~w_{i,2}. \\ \end{array} \end{aligned}$$

Intuitively, choosing one of the matchings (1)–(3) for the agents $m_{i,j},w_{i,j}$ corresponds to coloring vertex $v_i$ with one of the three colors in $\{ 1,2,3 \}$.

Then, for each edge $e = \{ v_{i_1},v_{i_2} \} \in E$, and for each color $c \in \{ 1,2,3 \}$, we will introduce a preference profile $P_{e,c}$ that ensures that in each certainly stable matching, it cannot be the case that both the agents $m_{i_1,j},w_{i_1,j}$ and the agents $m_{i_2,j},w_{i_2,j}$ are matched to each other according to matching (*c*). As a result, any certainly stable matching directly corresponds to a proper 3-coloring of *G*.

A detailed description of the preference profiles $P_0$ and $P_{e,c}$ and a proof of correctness for this reduction follows.

In $P_0$, for each  $i \in [n]$, the preferences for $m_{i,j},w_{i,j}$ are as follows:

$$\begin{aligned} m_{i,1}&: \quad w_{i,1},w_{i,2},w_{i,3},---&w_{i,1}&: \quad m_{i,2},m_{i,3},m_{i,1},--- \\ m_{i,2}&: \quad w_{i,2},w_{i,3},w_{i,1},---&w_{i,2}&: \quad m_{i,3},m_{i,1},m_{i,2},---\\ m_{i,3}&: \quad w_{i,3},w_{i,1},w_{i,2},---&w_{i,3}&: \quad m_{i,1},m_{i,2},m_{i,3},--- \end{aligned}$$

Observe that the matchings (1)–(3) are the only stable matchings restricted to the agents $\{m_{i,1},m_{i,2},m_{i,3},w_{i,1},w_{i,2},w_{i,3}\}$.

Next, we continue with the preference profiles $P_{e,c}$. Take an arbitrary $e = \{ v_{i_1},v_{i_2} \} \in E$ and an arbitrary $c \in \{ 1,2,3 \}$. In $P_{e,c}$, the preferences for $m_{i,j},w_{i,j}$ for each $i \in [n] {\setminus } \{ i_1,i_2 \}$ are exactly the same as in $P_0$. Only the preferences for $m_{i_1,j},w_{i_1,j}$ and $m_{i_2,j},w_{i_2,j}$ differ from $P_0$; namely, we construct these preferences as follows.

For $m_{i_1,j},w_{i_1,j}$, we start with preferences that (i) for all $m_{i_1,j}$ have  {$w_{i_1,1},w_{i_1,2},w_{i_1,3}$} as top three choices, (ii) for all $w_{i_1,j}$ have  {$m_{i_1,1},m_{i_1,2},m_{i_1,3}$} as top three choices, (iii) admit only matchings (1), (2), and (3) as stable matchings between the agents $m_{i_1,j},w_{i_1,j}$, and (iv) for the men $m_{i_1,j}$ the matching (*c*) is the worst option among the matchings (1), (2), and (3). Similarly, for $m_{i_2,j},w_{i_2,j}$, we start with preferences that satisfy conditions (i), (ii) and (iii), and additionally satisfy the condition (iv$'$) that for the women $w_{i_2,j}$ the matching (*c*) is the worst option among the matchings (1), (2), and (3). Then, we modify the preferences for $m_{i_1,1}$ and $w_{i_2,1}$ slightly. For $m_{i_1,1}$, we insert $w_{i_2,1}$ between his second and third preferred woman. Similarly, for $w_{i_2,1}$, we insert $m_{i_1,1}$ between her second and third preferred man. As a result, $m_{i_1,1}$ and $w_{i_2,1}$ form a blocking pair in this preference profile if both the agents $m_{i_1,j},w_{i_1,j}$ and the agents $m_{i_2,j},w_{i_2,j}$ are matched to each other using matching (*c*)— and not if either set of agents is matched to each other using some other matching $(c')$.

For example, consider $e = \{ v_{i_1},v_{i_2} \}$ and $c = 2$. The preferences for the agents $m_{i_1,j},w_{i_1,j}$ and $m_{i_2,j},w_{i_2,j}$ in the preference profile $P_{e,c}$ are as follows:

$$\begin{aligned} m_{i_1,1}&: \quad w_{i_1,1},w_{i_1,3},{\varvec{w_{i_2,1}}},w_{i_1,2},---&m_{i_2,1}&: \quad w_{i_2,2},w_{i_2,3},w_{i_2,1},---\\ m_{i_1,2}&: \quad w_{i_1,2},w_{i_1,1},w_{i_1,3},---&m_{i_2,2}&: \quad w_{i_2,3},w_{i_2,1},w_{i_2,2},---\\ m_{i_1,3}&: \quad w_{i_1,3},w_{i_1,2},w_{i_1,1},---&m_{i_2,3}&: \quad w_{i_2,1},w_{i_2,2},w_{i_2,3},---\\ w_{i_1,1}&: \quad m_{i_1,3},m_{i_1,2},m_{i_1,1},---&w_{i_2,1}&: \quad m_{i_2,1},m_{i_2,2},{\varvec{m_{i_1,1}}},m_{i_2,3},---\\ w_{i_1,2}&: \quad m_{i_1,1},m_{i_1,3},m_{i_1,2},---&w_{i_2,2}&: \quad m_{i_2,2},m_{i_2,3},m_{i_2,1},---\\ w_{i_1,3}&: \quad m_{i_1,2},m_{i_1,1},m_{i_1,3},---&w_{i_2,3}&: \quad m_{i_2,3},m_{i_2,1},m_{i_2,2},--- \end{aligned}$$

We argue that *G* has a proper 3-coloring if and only if there is a certainly stable matching for the probability distribution over preference profiles that we constructed.

$(\Rightarrow )$ Firstly, suppose that *G* has a proper 3-coloring, say $\chi : V \rightarrow \{ 1,2,3 \}$. We can then construct a certainly stable matching as follows. For each  $i \in \{1,\dots ,n\}$, we match the agents $m_{i,j},w_{i,j}$ to each other using matching  $(\chi (v_i))$. Clearly, this matching is stable for $P_0$. Moreover, because $\chi$ is a proper 3-coloring of *G*, it is straightforward to verify that this matching is also stable for each $P_{e,c}$.

$(\Leftarrow )$ Conversely, suppose that there is a certainly stable matching. We know that in this matching, each man $m_{i,j}$ must be matched to some woman $w_{i,j'}$, and vice versa, each woman $w_{i,j}$ must be matched to some man $m_{i,j''}$. If this were not the case, the matching would not be stable for $P_0$, and thus not certainly stable. Moreover, by a similar argument, we know that for each  $i \in \{1,\dots ,n\}$, the matching between the men $m_{i,j}$ and the women $w_{i,j}$ must be one of the matchings (1), (2), or (3). We can then construct a 3-coloring $\chi : V \rightarrow \{ 1,2,3 \}$ as follows. For each  $i \in \{1,\dots ,n\}$, we set $\chi (v_i)$ in such a way that $(\chi (v_i))$ is the matching used in the certainly stable matching to match the men $m_{i,j}$ and the women $w_{i,j}$ to each other.

We argue that $\chi$ is a proper 3-coloring of *G*. Suppose that this is not the case, that is, that there is some $e = \{ v_{i_1},v_{i_2} \}$ such that $\chi (v_{i_1}) = \chi (v_{i_2}) = c$. Now consider the preference profile $P_{e,c}$. By construction of $\chi$, we know that in the certainly stable matching, agent $m_{i_1,j}$ is matched to agent $w_{i_1,j}$ and agent $m_{i_2,j}$ is matched to agent $w_{i_2,j}$, both times according to matching (*c*). However, by construction of $P_{e,c}$ we will have that  $m_{i_1,1}$ and $w_{i_2,1}$ form a blocking pair in $P_{e,c}$. This is a contradiction with our assumption that the matching we considered is certainly stable. From this, we can conclude that $\chi$ is a proper 3-coloring of *G*. $\square$

By reducing from 3-Coloring on 4-regular graphs and using Vizing’s theorem to combine preference profiles, we can strengthen Theorem 11 and show that the result holds even when the input contains only a constant number of preference profiles.

### Corollary 6

For the joint probability model, ExistsCertainlyStableMatching is NP-complete, even when there are only 16 preference profiles in the lottery.

### Proof

We show this by modifying the proof of Theorem 11. We know that 3-Coloring is NP-complete even when restricted to 4-regular graphs [8]. We use the reduction in the proof of Theorem 11, and we assume that in the given graph *G*, each vertex has degree 4. Then, by Vizing’s Theorem [28], we know that we can give a proper edge coloring of *G* that uses at most 5 colors. Moreover, we can find such an edge coloring in polynomial time. Then, since in the proof of Theorem 11, in each preference profile $P_{e,c}$ with $e = \{ v_{i_1},v_{i_2} \}$, only the preferences for the agents $m_{i_1,j},w_{i_1,j},m_{i_2,j},w_{i_2,j}$ differ from $P_0$, we can, for each color $c \in \{ 1,2,3 \}$, combine the preference profiles $P_{e,c}$ for all edges *e* that are colored with the same color. This results in only 16 preference profiles: $P_0$, and a preference profile for each of the 5 edge colors and each of the 3 vertex colors. $\square$

## Future work

First we note that we left open two outstanding questions, as described in Table 3. In this paper we focused on the problem of computing a matching with the highest stability probability. However, a similarly reasonable goal could be to minimize the expected number of blocking pairs. It would also be interesting to investigate some further realistic probability models, such as the situation when the candidates are ranked according to some noisy scores (like the SAT scores in the US college admissions). This would be a special case of the joint probability model that may turn out to be easier to solve.

One may also consider the questions we asked in this paper for two natural generalizations on the one-to-one matching problem: many-to-one and the many-to-many two-sided problems, where the agents on one or both sides of a two-sided market have capacities. These are reasonable extensions with regard to many applications, e.g., college admissions, and course allocation, respectively. One issue when considering capacities larger than one is that there are many ways of extending the preferences over candidates to preferences over sets of candidates. Another possible generalization of the one-to-one marriage model is the non-bipartite stable roommates problem. We conjecture that most of our polynomial tractability results can be extended to these more general models, but we leave this work for future research.

## Footnotes

1 We note that the complexity of all problems that we study is the same for complete and incomplete lists, where non-listed agents are deemed unacceptable—see Proposition 1 in Sect. 3.1.
